# Serum levels of retinol-binding protein-4 are associated with the presence and severity of coronary artery disease

**DOI:** 10.1186/s12933-014-0121-z

**Published:** 2014-08-21

**Authors:** Vaia Lambadiari, Nikolaos PE Kadoglou, Vassilios Stasinos, Eirini Maratou, Aias Antoniadis, Fotios Kolokathis, John Parissis, Erifili Hatziagelaki, Efstathios K Iliodromitis, George Dimitriadis

**Affiliations:** 2nd Department of Internal Medicine, Research Institute and Diabetes Centre, Athens University Medical School, Attikon University General Hospital, 1st Rimini Street, GR-12462 Haidari, Greece; 2nd Department of Cardiology, Athens University Medical School, Attikon University General Hospital, 1st Rimini Street, GR-12462 Haidari, Greece; Hellenic National Centre for Research, Prevention and Treatment of Diabetes Mellitus and its Complications, Ploutarchou Street, GR −10675 Athens, Greece

**Keywords:** Retinol-Binding Protein-4, Myocardial infarction, Coronary artery disease, Adipokines

## Abstract

**Background:**

The interplay between the novel adipokine retinol-binding protein-4 (RBP4) and coronary artery disease (CAD) is still obscure. We investigated the relationship between RBP4 levels and the presence and severity of angiographically proven CAD and determined its possible role in acute myocardial infarction (AMI).

**Methods:**

305 individuals with angiographically proven CAD (CAD-patients), were classified into 2 subgroups: 1) acute myocardial infarction (AMI, n = 141), and 2) stable angina (SA, n = 164). Ninety-one age- and sex-matched individuals without CAD, but with at least 2 classical cardiovascular risk factors, served as controls (non-CAD group). RBP4 serum levels were measured at hospital admission and were analyzed in relation to the coronary severity stenosis, assessed by the Gensini-score and the number of coronary narrowed vessels. Other clinical parameters, including insulin levels, HOMA-IR, hsCRP, glycaemic and lipid profile, and left-ventricular ejection fraction were also assessed.

**Results:**

Serum RBP4 levels were significantly elevated in patients with CAD compared to non-CAD patients (39.29 ± 11.72 mg/L vs. 24.83 ± 11.27 mg/L, p < 0.001). We did not observe a significant difference in RBP4 levels between AMI and SA subgroups (p = 0.734). Logistic regression analysis revealed an independent association of CAD presence with serum RBP4 (β = 0.163, p = 0.006), and hsCRP (β = 0.122, p = 0.022) levels, in the whole study group. Among variables, hsCRP (β = 0.220), HDL (β = −0.150), and RBP4 (β = 0.297), correlated in both univariate and multivariate analysis with CAD severity (R^2^ = 0.422, p < 0.001). Similarly, RBP4 concentrations increased with the number of coronary narrowed vessels (p < 0.05).

**Conclusion:**

Patients with CAD, both SA and AMI, showed elevated RBP4 serum levels. Notably, increased RBP4 concentration seemed to independently correlate with CAD severity, but no with AMI.

**Trial registration:**

The ClinicalTrials.gov Identifier is: NCT00636766

## Introduction

Coronary artery disease (CAD) is the leading cause of morbidity and mortality worldwide with the risk for CAD development being higher among patients with obesity, diabetes and other insulin resistant states. Numerous studies have been previously conducted in order to identify novel biomarkers indicating either the presence or the severity of CAD [1,2]. Such a precise cardiovascular risk assessment is crucial for the choice of diagnostic methods and intensive therapeutic modalities (e.g. coronary angiography) with obvious benefits and risks [3]. Thus, the identification of new serum biomarkers would add to the prognostic value of the traditional ones.

A growing body of evidence, the adipocyte-derived cytokines, known as adipokines, seem to interfere with the crosstalk between adipose tissue, insulin resistance and CAD [4]. Retinol-Binding Protein-4 (RBP4), a novel adipokine/hepatokine, initially identified by Yang et al. [5], is the main transport protein for retinol (vitamin A) in the circulation. Most, but not all studies, have demonstrated its link with insulin resistance and obesity [5–7]. Elevated plasma RBP4 levels have been predominantly found in men and women with abdominal adiposity even in the non-diabetic state [8,9]. Similarly, high RBP4 levels in obese children seem to be related to adipose tissue mass, to the differentiation of adipocytes, and to multiple risk factors for adiposity-related co-morbidities [10,11].

Accumulating data strongly support the association of RBP4 circulating levels with traditional, (e.g. dyslipidaemia, hypertension, albuminuria) and non-traditional cardiovascular risk factors (e.g. cytokines) mainly through the impairment of glucose and lipid metabolism and adipose tissue dysfunction, despite that opposite findings put RBP4 changes in dispute [12–14]. Although, the involvement of RBP4 in the development of subclinical atherosclerosis has been proven [15], its prognostic value in carotid [16] or coronary [17] atherosclerosis progression is still obscure. This raises the question whether circulating RBP4 concentrations could serve as a CAD predictor. Regarding the aforementioned inconsistent interplay of RBP-4 with diabetes and obesity, it becomes more valuable to assess RBP4 levels among diabetic and non-diabetic cohorts.

In this study, we sought to investigate the relationship between RBP4 levels and the presence and severity of angiographically proven CAD, and to determine its possible role in acute myocardial infarction (AMI).

## Methods

### Study population

We initially identified 868 individuals who underwent coronary angiography from January to June 2012 in “Attikon” University Hospital of Athens, Greece. We then excluded patients with history of recent, −within the past 6 months- severe chronic heart failure (class NYHA II - IV), malignant diseases, major trauma or surgery, severe renal (creatinine > 2 mg/dl) or liver insufficiency (ALT > 2 times upper normal limit), acute or chronic infectious disease, or any kind of immune-mediated disease. Among individuals undergoing coronary angiography, 305 patients with significant, angiographically proven CAD, defined as stenosis of more than 50% of the luminal diameter in a major epicardial coronary vessel, were assigned to the CAD-group. Based on clinical and laboratory findings, the latter group was further subdivided into the following subgroups:

1. AMI (n = 141): Patients consecutively hospitalized in the coronary care unit of our department with acute myocardial infarction (AMI) (STEMI, NSTEMI) within 12 hours of symptoms onset. The AMI diagnosis was made on the basis of typical symptoms consistent with myocardial ischemia (chest discomfort or anginal equivalent) that continued for >30 min, newly developed ischemic ST-T changes (ST-elevation or ST-segment depression or prominent T-wave inversion) in at least 2 contiguous ECG leads, and elevated cardiac-associated biomarkers of necrosis in an appropriate clinical setting.
2. SA (n = 164): Consecutive patients with stable angina (SA) diagnosed as: chest pain or angina-equivalent on exertion, signs of myocardial ischemia on functional testing (myocardial perfusion scintigraphy, stress echocardiography, exercise electrocardiography) and established CAD defined as previous myocardial infarction or percutaneous coronary intervention or coronary artery bypass graft. We excluded patients with unstable angina, such as those with recent onset of cardiac-origin symptoms or angina at rest and normal levels of cardiac troponin.

Among the remaining individuals without angiographically proven CAD, we selected a cohort of 91 individuals, age- and sex-matched to CAD group. That group had at least 2 cardiovascular risk factors (diabetes, dyslipidemia, hypertension, obesity, positive family history for early CAD), but no clinical evidence of CAD, and served as control (non-CAD group). The main purpose to set such selection criteria in the control group was to limit confounders and create more comparable groups.

The medical history of all participants was comprehensively recorded, and a clinical examination, electrocardiogram (ECG), trans-thoracic echocardiography and blood sampling, were carried out before angiography. The study complied with the Declaration of Helsinki, and the trial protocol was approved by the local Ethics Committee. Written informed consent was obtained from all patients before entering the study.

### Clinical and echocardiographic examination

At the time of a clinical examination, body mass index (BMI, calculated as weight (kg) / height (m)^2^), waist-hip ratio (WHR), blood pressure, were determined. Waist circumference was assessed at the level midway between the lower rib margin and the iliac crest. The hips were measured at the level of the greater femoral trochanters. Thus, WHR expressed waist circumference divided by hip girth. BP was measured twice, after keeping all participants in a sitting position for 15 minutes. There was a 5 minute interval between the two measurements and the mean value was estimated for study purposes. Medications, co-morbidities and smoking history were assessed through a structured questionnaire preceding clinical examination. Before angiography, we also obtained the patients’ regular prescribed medications, based on medical records of the attending physicians. Diabetes was considered present if a patient was treated with insulin or oral agents or had a fasting glucose level ≥126 mg/dl (7.0 mmol/L), HbA1c ≥6.5% or a known history of diabetes. Hypertension was defined by systolic blood pressure ≥140 mmHg, diastolic blood pressure ≥90 mmHg, the current use of antihypertensive treatment, or a combination of the 3. Dyslipidemia was defined as non-HDL levels ≥190 mg/dl, the current use of lipid-lowering treatment, or both [18]. Echocardiographic examination (Vivid 7; General Electric, Ohio, OH, USA) was performed in all participants by the same operator in order to evaluate left ventricular morphology and systolic function.

### Coronary angiography

All participants underwent coronary angiography by experienced cardiologists. Coronary artery severity was assessed using the number of narrowed vessels (>50%) Gensini score, which is derived from the co-evaluation of the number of stenotic coronary artery segments, the degree of their lumen stenosis and the localization of stenotic change [4]. The Gensini score was given as 1 for 1–25% narrowing, 2 for 26–50% narrowing, 4 for 51–75% narrowing, 8 for 76–90% narrowing, 16 for 91–99% narrowing and 32 for total occlusion. The score is then multiplied by a factor that takes into account the importance of the lesion's position in the coronary arterial tree, for example, 5 for the left main coronary artery, 2.5 for the proximal left anterior descending coronary artery (LAD) or proximal left circumflex coronary artery (LCX), 1.5 for the mid-region of the LAD, 1.5 for proximal and mid-region RCA, and 1 for the distal LAD, mid-distal region of the LCX and RCA. The average score for all the coronary arteries expressed Gensini score [4].

### Blood analyses

In patients with AMI, blood samples were drawn on admission and before treatment initiation to assess inflammatory markers. The rest of biochemical parameters, such as lipids and glycaemic indexes (fasting plasma glucose – FPG, glycosylated haemoglobin – HbA1c) were assayed after an overnight fast at least 24 h after admission. Similarly, venous blood samples were collected following an overnight fasting, in the rest of groups (SA and non-CAD). Glucose and lipids were analysed enzymatically (Chemwell 2910; Awareness Technology Inc, Palm City, Fl, USA). Low density lipoprotein cholesterol (LDL-C) was calculated by Friedewald formula. The intra-assay coefficients of variation (CVs) for total cholesterol, triglycerides, HDL and FPG were: 0.84%, 0.4%, 1.57% and 0.6%, respectively. In parallel, the inter-assay CVs for the abovementioned parameters were 1.3%, 1.3%, 2.00%, and 1.6%, respectively. The HbA1c was determined by high-performance liquid chromatography (Menarini Diagnostics, Florence, Italy) in the diabetic subpopulation. A white blood cells (WBC) count analysis was performed using Cell Dyne 1700 electronic counter (Sequoia-Turner Corporation, Santa Clara, CA, USA), with 1.99% intra-assay CV and 1.92% inter-assay CV.

To measure insulin and RBP4, serum was separated from the blood corpuscles by centrifugation at 5000 g for 10 min and kept frozen at −80°C until the analysis. We quantified serum insulin with IRMA kit (DIAsource ImmunoAssays S.A., Louvain-la-Neuve, Belgium). The inter- and intra-assay CVs were 6.3% and 2%, respectively. Insulin resistance was calculated by a homeostasis-model-assessment (HOMA-IR) index with the following formula: HOMA-IR = fasting insulin (mU/L) × FPG (mg/dl)/405. Using a commercially available enzyme immunoassay kit, we assayed serum concentrations of RBP4 (Immunodiagnostik AG, Bensheim, Germany). The intra-assay CV was 9.7% and the inter-assay CV was 5%. Measurement of high-sensitivity CRP (hsCRP) was performed using a particle enhanced immunoturbidimetric assay (Hitachi 917 analyser; Boehringer Mannheim, Germany). The detection limit was 0.1 mg/L, with intra- and inter-assay CVs of 1.34% and 5.7%, respectively. All serum concentrations (triplicate determinations) were analysed in a blinded manner with respect to any clinical information.

### Statistical methods

Results are presented as mean values ± standard deviation (SD). Normality of distribution was assessed with Kolmogorov-Smirnov test. We used student’s t-test and chi-square for comparison of parametric and non-parametric variables between CAD versus non-CAD group and AMI versus SA subgroup. Comparison across all cohorts (AMI, SA and non-CAD) was performed by one-way ANOVA and post-hoc Tuckey analysis. The relationships of RBP4 with other variables and CAD severity indexes were evaluated with Pearson correlation and standard multiple regression analysis. We explored the independent determinants of CAD in the whole study group using logistic regression analysis and the receiver operating characteristic (ROC) curve. A two-tailed p value <0.05 was considered to be statistically significant. We used the computer statistical software package SPSS (version 17.0; SPSS Inc, Chicago, IL, USA).

## Results

### Clinical and biochemical variables

The clinical and laboratory characteristics of the whole study cohort and CAD subgroups are presented in Tables 1 & 2, respectively. Between CAD and non-CAD groups we observed significant (p < 0.05) differences in smoking and dyslipidemia rates, glycemic control, SBP, HDL, WBC, insulin resistance and hsCRP levels. Subgroup analysis showed that most of the aforementioned differences between CAD and non-CAD groups were predominantly driven by the acute phase of AMI subgroup. Thereby, those differences disappeared between SA and non-CAD patients (p > 0.05), except WBC and hsCRP. As expected, CAD group had higher prescription rate of statins and anti-platelet agents compared to non-CAD (p < 0.001).

**Table 1:** **Clinical and biochemical parameters, angiographic findings and echocardiographic estimation of left ventricular ejection fraction in CAD and non-CAD**

	***CAD group*** ***(n = 305)***	***Non-CAD group*** ***(n = 91)***	***P value***
**Males, n (%)**	269 (88.20)	74 (81.32)	0.229
**Age (y)**	64 ± 13	62 ± 11	0.563
**Smoking, n (%)**	149 (48.85)	23 (25.27)	<0.001
**Hypertension, n (%)**	195 (63.93)	61 (67.03)	0.109
**Dyslipidemia, n (%)**	253 (82.95)	61 (67.03)	0.049
**Diabetes, n (%)**	74 (24.26)	16 (17.58)	0.184
**Medications**			
**Statins, n (%)**	159 (52.13)	25 (27.47)	<0.001
**Fibrates, n (%)**	6 (1.97)	2 (2.2)	0.902
**ACEIs/ARBs, n (%)**	152 (49.83)	48 (52.75)	0.891
**Anti-platelets, n (%)**	178 (58.36)	15 (16.48)	<0.001
**BMI (kg/m** ^**2**^ **)**	28.93 ± 4.60	27.71 ± 4.22	0.088
**WHR**	0.97 ± 0.11	0.95 ± 0.09	0.133
**SBP (mmHg)**	145 ± 23	130 ± 17	<0.001
**DBP (mmHg)**	81 ± 13	80 ± 12	0.310
**TChol (mg/dl)**	193 ± 46	201 ± 52	0.401
**HDL-C (mg/dl)**	44 ± 13	48 ± 13	<0.001
**LDL-C (mg/dl)**	121 ± 43	128 ± 43	0.398
**TG (mg/dl)**	142 ± 77	132 ± 66	0.280
**FPG (mg/dl)**	135 ± 67	120 ± 25	0.045
HbA1c (%)*	7.7 ± 1.1	7.1 ± 0.9	0.020
**Insulin (mU/L)**	15.03 ± 8.71	9.01 ± 4.73	0.004
**HOMA-IR**	5.01 ± 2.45	2.67 ± 0.72	0.001
**WBC (cells/μL)**	9489 ± 3384	6826 ± 1858	<0.001
**hsCRP (mg/L)**	9.24 ± 3.16	2.97 ± 1.28	<0.001
**RBP-4 (mg/L)**	39.29 ± 11.72	24.83 ± 11.27	<0.001
**LVEF (%)**	54 ± 9	58 ± 9	0.009
**Angiography**			
**1-vessel**	126 (41.31)	-	-
**2-vessels**	131 (42.95)	-	-
**3-/4-vessels**	48 (15.74)	-	-

Data are expressed as means ± SD. n, number of patients; ACEIs, Angiotensin-converting enzyme inhibitors; ARBs, Angiotensin II Receptor Blockers; BMI, body-mass index; WHR, waist-hip ratio; SBP, systolic blood pressure, DBP, diastolic blood pressure; TChol, total cholesterol; TG, triglycerides; FPG, fasting plasma glucose; HOMA-IR, homeostasis model assessment; WBC, white blood cells; hsCRP, high-sensitivity CRP; LVEF, left ventricular ejection fraction.

*HbA1c was measured only in the diabetic subgroup.

**Table 2:** **Clinical and biochemical parameters, angiographic findings and echocardiographic estimation of left ventricular ejection fraction within CAD group**

	***AMI subgroup*** ***(n = 141)***	***SA subgroup*** ***(n = 164)***	***P value***
**Males, n (%)**	114 (80.85)	150 (91.46)	0.541
**Age (y)**	63 ± 13	66 ± 10	0.204
**Smoking, n (%)**	94 (66.67)	55 (33.54)	<0.001
**Hypertension, n (%)**	93 (65.96)	102 (62.20)	0.702
**Dyslipidemia, n (%)**	106 (75.18)	147 (89.63)	0.403
**Diabetes, n (%)**	27 (19.14)	47 (28.66)	0.113
**Medications**			
**Statins, n (%)**	28 (19.86)	141 (85.97)	<0.001
**Fibrates, n (%)**	3 (2.13)	3 (1.83)	0.799
**ACEIs/ARBs, n (%)**	59 (41.84)	86 (52.44)	0.188
**Anti-platelets, n (%)**	20 (14.18)	158 (96.34)	<0.001
**BMI (kg/m** ^**2**^ **)**	28.29 ± 4.43	29.48 ± 5.03	0.085
**WHR**	0.97 ± 0.11	0.98 ± 0.09	0.356
**SBP (mmHg)**	149 ± 24	141 ± 19	0.018
**DBP (mmHg)**	84 ± 14	78 ± 11	0.007
**TChol (mg/dl)**	210 ± 47	178 ± 42	0.038
**HDL-C (mg/dl)**	44 ± 12	45 ± 13	0.942
**LDL-C (mg/dl)**	141 ± 44	104 ± 36	<0.001
**TG (mg/dl)**	125 ± 45	145 ± 83	0.082
**FPG (mg/dl)**	148 ± 70	124 ± 55	0.032
HbA1c (%)*	8.1 ± 1.3	7.6 ± 1.2	0.048
**Insulin (mU/L)**	15.26 ± 4.97	14.83 ± 5.12	0.861
**HOMA-IR**	5.58 ± 2.04	4.54 ± 1.95	0.223
**WBC (cells/μL)**	9912 ± 3526	8117 ± 2515	<0.001
**hsCRP (mg/L)**	13.04 ± 5.68	5.97 ± 1.03	<0.001
**RBP-4 (mg/L)**	37.83 ± 17.34	38.76 ± 11.36	0.734
**LVEF (%)**	54 ± 8	55 ± 9	0.531
**Angiography**			
**1-vessel**	71 (50.35)	51 (31.09)	0.205
**2-vessels**	38 (26.95)	55 (33.54)	0.743
**3-/4-vessels**	32 (22.70)	58 (35.37)	0.063

Data are expressed as means ± SD. AMI, acute myocardial infarction; SA, stable angina; n, number of patients; ACEIs, Angiotensin-converting enzyme inhibitors; ARBs, Angiotensin II Receptor Blockers; BMI, body-mass index; SBP, systolic blood pressure, DBP, diastolic blood pressure; TChol, total cholesterol; TG, triglycerides; FPG, fasting plasma glucose; HOMA-IR, homeostasis model assessment; WBC, white blood cells; hsCRP, high-sensitivity CRP; LVEF, left ventricular ejection fraction.

*HbA1c was measured only in the diabetic subgroup.

The AMI subgroup showed higher smoking rate (p < 0.001), SBP (p = 0.018) and DBP (p = 0.007) levels compared to SA subgroup. Regarding medications, we observed lower usage of statins (p < 0.001) and antiplatelet agents (p < 0.001) in AMI that SA subgroup before angiography. Notably, no patient was receiving vitamin supplements, which might have affected our results. As long as it concerns biochemical parameters, AMI-patients appeared with worse lipid profile and increased levels of FPG, HbA1c, WBC and hsCRP than the SA subgroup (p < 0.05).

Significantly higher RBP4 values were found in CAD rather than non-CAD patients (p < 0.001). Importantly, RBP4 concentrations did not differ between AMI and SA subgroups (p = 0.734). However, both AMI and SA subgroups showed considerably upregulated RBP4 concentrations than non-CAD group (p = 0.010, p < 0.001, respectively) (Tables 1 & 2). Further subgroup analysis did not reveal any influence of gender, diabetes or statins’ usage on RBP4 levels within both CAD and non-CAD groups (p > 0.05).

### Correlations

We searched for correlations of RBP4 with the rest of variables within the CAD group. In univariate analysis, RBP4 significantly correlated with hsCRP, fasting insulin and HOMA-IR. All those correlations remained significant in multivariate analysis (hsCRP: β = 0.190, p = 0.037; fasting insulin: β = 0.331, p < 0.001; HOMA-IR: β = 0.326, p < 0.001) (R^2^ = 0.380, p < 0.001).

In univariate analysis, the severity of CAD, quantified by the Gensini score, was significantly correlated with FPG, HOMA-IR, hsCRP, HDL and RBP4 (p < 0.05). The latter variables entered standard multiple regression analysis (Table 3). Among variables, hsCRP, HDL and RBP4 remained as independent determinants of the Gensini score (R^2^ = 0.422, p < 0.001). In addition to this, RBP4 levels significantly increased across the number of diseased vessels from 34.19 ± 17.3 mg/L (1-vessel group) to 41.56 ± 16.32 mg/L (2-vessels group) and 49.19 ± 17.3 mg/L (3-/or 4-vessels group) (p < 0.05). In case of hsCRP, we found significant difference between 1-vessel and 3-/or 4-vessels group (7.02 ± 2.18 mg/dl vs 12.57 ± 3.99 mg/dl, p = 0.011). On the other hand, HDL levels did not significantly change across the number of narrowed vessels.

**Table 3:** **Standard multiple regression analysis of Gensini score (dependent variable) and other independent variables**

	***Gensini score***		
	***β***	***95% CI***	***P value***
**RBP4**	0.297	0.028 – 0.587	0.012
**HDL-C**	−0.150	−0.205 – 0.001	0.040
**FPG**	0.121	−0.002 – 0.303	0.269
**HOMA-IR**	0.038	−0.042 – 0.059	0.745
**hsCRP**	0.220	0.006 – 0.370	0.004

**Figure 1:**
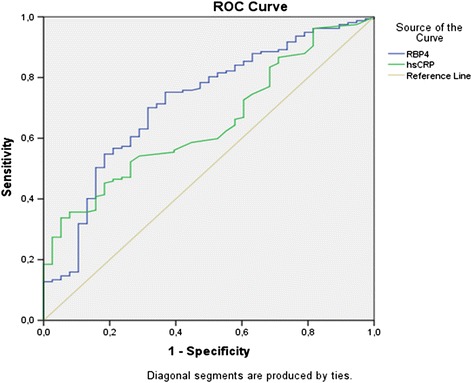
**Receiver operating characteristic curve analysis of Retinol-Binding Protein-4 (RBP4) (blue line) and hsCRP (green line) as markers for the diagnosis of coronary artery disease (CAD).**

Multiple logistic regression analysis was performed in the whole study group to estimate the association of significant CAD presence with clinical and biochemical variables, after adjustment for traditional cardiovascular risk factors like diabetes, hypertension, dyslipidemia, smoking and male gender. The presence of significant CAD was independently associated with serum RBP4 (β = 0.163, p = 0.006), and hsCRP (β = 0.122, p = 0.022) levels. The ROC curves of RBP4 and hsCRP for the discrimination between CAD or not are shown in Figure 1. Using ROC analysis, the area under the curve (AUC) was 0.719 (95% CI 0.632-0.805, p < 0.001) for RBP4 and 0.649 (95% CI 0.561-0.736, p = 0.005) for hsCRP.

## Discussion

The present cross-sectional study demonstrated increased serum RBP4 levels in patients with CAD (either AMI or SA) compared to age- and sex-matched individuals without CAD, but with at least 2 classical cardiovascular risk factors. In our study cohort, the presence of significant CAD was independently related to RBP4 and hsCRP circulating levels. This is the first study demonstrating the independent association of RBP4 with CAD severity indexes (e.g. Gensini score and number of narrowed vessels).

In spite of the rather established relation to insulin resistance and cardiovascular risk factors, the actual association of RBP4 levels with atherosclerotic-related cardiovascular disease is still controversial. Previous studies have shown the positive association of RBP4 with subclinical atherosclerosis [19–21]. In elderly men, RBP4 levels correlated with hypertriglyceridemia and prior cerebrovascular disease [22]. Furthermore, plasma RBP4 concentration proved to be related to the presence of cardiovascular disease in non-obese, non-diabetic subjects [23]. A recent prospective trial suggested its value in predicting CAD in a large women cohort during a follow-up period of 9 to 16 years [24]. In contrast, another large prospective trial doubted the additive prognostic value of RBP4 among CAD-free men and women [17]. Further recent studies in experimental models of high cardiovascular risk in humans have actually exhibited a protective role of RBP4 in vasculature [25,26], or even reduced levels in men with AMI [27], which add to the controversy concerning the actual role of RBP4 in atherosclerosis. However, in these studies the samples are smaller, they mainly include men, and do not distinguish between subjects according to the actual vessel pathology but rather according to cardiovascular risk. In our study cohort, consisting of patients with SA, AMI and non-CAD subjects, RBP4 and hsCRP levels were independently associated with the presence of significant CAD. It is worth mentioning that the non-CAD patients had at least two cardiovascular risk factors, presented with high suspicion of CAD. Thus, this is the first study implicating the potential of serum RBP4 levels in discriminating patients with established CAD from high risk patients, using coronary angiographic criteria. Notably, the presence of AMI did not affect RBP4 serum levels among CAD patients, implicating the dissociation of RBP4 from acute coronary event. Perhaps, circulating RBP4 is predominantly influenced by the presence of coronary atherosclerotic lesions rather than the atherosclerotic plaque destabilization. However, this postulation needs further investigation.

Accumulating data support the relationship of novel adipokines with CAD severity [28]. To our knowledge this is the first study indicating the association of CAD severity, expressed by Gensini score, with RBP4 levels in addition to hsCRP and HDL. Interestingly, RBP4 levels significantly increased across the number of diseased vessels. Those striking findings suggest an interaction between RBP4 and the pathophysiological process of coronary atherosclerosis. The latter notion has been recently supported by the higher RBP4 expression in epicardial fat derived from CAD rather than non-CAD patients [29]. Those authors hypothesized higher protein released in close proximity to coronary arteries, implying a causative role in the pathogenesis of coronary atherosclerosis. Perhaps, measuring serum RBP4 could contribute to patients’ risk stratification in order to avoid diagnostic procedure that bares risks itself, such as coronary angiography. Unambiguously, future trials will clarify the emerging role of serum RBP4 as a valid biomarker of CAD extent.

Regarding the underlying mechanisms, we observed the independent correlation of RBP4 with insulin resistance indices and established markers of inflammation, like hsCRP. More recently, RBP4 levels independently predicted early endothelial dysfunction, linking adipose tissue inflammation and subclinical atherosclerosis in non-diabetic individuals [30]. The association of RBP4 with markers of inflammation is supported by several studies. RBP4 was found to induce *in vitro* inflammation in endothelial cells, by stimulating expression of proinflammatory molecules, such as vascular cell adhesion molecule 1 (VCAM-1), E-selectin, intercellular adhesion molecule 1 (ICAM-1), monocyte chemoattractant protein 1 (MCP-1), and interleukin-6 (IL-6) [31]. Those effects may be mediated via the activation of NADPH oxidase and NF-κB leading to endothelial inflammation. In another study involving patients with diabetes and CAD, RBP4 levels rose in subjects with both conditions, and were rather correlated with TNFa than with markers of insulin resistance [32].

The role of retinoids in lipid metabolism is well known and is mediated through the regulation of ApoC-III and VLDL production and fatty acid oxidation [33]. A relatively large study of patients with type 2 diabetes or CAD previously reported the relation of RBP4 levels to an unfavorable lipid profile [34]. In the diabetic state, a positive association of RBP4 with plasma triglycerides levels and VLDL-apoB100 total fractional catabolic rate has also been found [35]. The latter evidence suggests a potential interaction between RBP4 and CAD through pro-atherogenic lipoproteins and their enzymes. Moreover, RBP4 has been recently identified as an HDL-associated protein; it is demonstrated that in patients with acute coronary syndrome, HDL shifts to an inflammatory profile, which can in turn, alter the protective effects of HDL on the atherosclerotic plaque. Thus, in this inflammatory milieu, RBP4 could also share such properties [36]. In parallel, RBP4 has exhibited modest heritability and sexual dimorphism (higher levels in men) [37], while it is considered to represent a link between visceral adiposity and cardiovascular disease [9]. Taken together, our study failed to reveal any association of RBP4 with lipids, gender or BMI. Perhaps, the lipid-lowering medications, the low percentage of women and the vast majority of overweight, but non-obese participants, might have confounded the relationship of the above parameters, respectively, with RBP4 levels.

The major limitation of the present investigation was the cross-sectional design, which prevented us from inferring cause-effect relationship of RBP4 with CAD. Although we did not recognise differences between acute and stable condition of CAD, the cross-sectional design of our study did not allow us to evaluate the association of RBP4 with either AMI occurrence or long-term clinical outcomes. Since the majority of patients with classical cardiovascular risk factors (e.g. diabetes, dyslipidemia, hypertension etc.) were already treated, we cannot rule out the plausible effects of pharmaceutical agents (e.g. statins) on RBP4, leading to underestimation of its predictive power. Another important limitation was the considerable differences in some biochemical parameters between CAD and non-CAD groups, which might have affected RBP4 fluctuations. Despite the independent association between RBP4CAD and CAD diagnosis, the absence of matching for baseline characteristics may weaken our conclusions. Finally, as our control group sample comprised of patients with cardiovascular risk factors, we couldn’t extrapolate our conclusions to healthy subjects. Another study limitation is the potential influence of the transthyretin TTR-RBP4 complex in the affinity towards RBP4 that could be interfering with the ELISA measurement of RBP4. However, a previous study has shown that circulating RBP4 and TTR were not affected by human obesity or T2DM, compared to lean controls [38]. The same could apply to our population as well, considering that a low grade inflammation often coexists with T2DM and obesity and that in our study we did not recognize differences between acute and stable condition of CAD.

## Conclusion

In conclusion, the present study documented RBP4 being a strong predictor of CAD, defined as angiographically significant coronary stenosis. That result was not influenced by acute (AMI) or stable (SA) CAD phase. Most importantly, RBP4 levels seemed to independently correlate to CAD severity. Thus, RBP4 could be a cost-effective, easy to obtain, novel risk biomarker, that could contribute to improved clinical decision making and management of patients at risk of CAD.
